# Cancer metabolic reprogramming: importance, main features, and potentials for precise targeted anti-cancer therapies

**DOI:** 10.7497/j.issn.2095-3941.2014.01.001

**Published:** 2014-03

**Authors:** Liem Minh Phan, Sai-Ching Jim Yeung, Mong-Hong Lee

**Affiliations:** ^1^Department of Molecular and Cellular Oncology, ^2^Department of General Internal Medicine, Ambulatory Treatment and Emergency Care, ^3^Department of Endocrine Neoplasia and Hormonal Disorders, The University of Texas MD Anderson Cancer Center, 1515 Holcombe Blvd, Houston, Texas, TX77030, USA

**Keywords:** Cell cycle, energy metabolism, glycolysis, glutaminolysis, mitochondria biogenesis

## Abstract

Cancer cells are well documented to rewire their metabolism and energy production networks to support and enable rapid proliferation, continuous growth, survival in harsh conditions, invasion, metastasis, and resistance to cancer treatments. Since Dr. Otto Warburg’s discovery about altered cancer cell metabolism in 1930, thousands of studies have shed light on various aspects of cancer metabolism with a common goal to find new ways for effectively eliminating tumor cells by targeting their energy metabolism. This review highlights the importance of the main features of cancer metabolism, summarizes recent remarkable advances in this field, and points out the potentials to translate these scientific findings into life-saving diagnosis and therapies to help cancer patients.

## Cancer metabolism: major remodeling of cellular energy production and metabolic pathways in tumors

Cancer metabolic reprogramming has been recognized as one of the ten cancer hallmarks by Drs. Hanahan and Weinberg in their seminal review paper published in 2011^1^. Some of the most striking changes of tumor cellular bioenergetics include elevation of glycolysis, increase in glutaminolytic flux, upregulation of amino acid and lipid metabolism, enhancement of mitochondrial biogenesis, induction of pentose phosphate pathway and macromolecule biosynthesis^1^^-^^17^.

### Glycolysis

Compared to normal cells, cancer cells prefer using glycolysis even in normoxic condition^18^^-^^20^. This phenomenon is often referred as the Warburg effect because Dr. Otto Warburg discovered and reported these metabolic alterations in tumors in 1930 and 1956^18^^-^^20^. Many decades later, numerous studies have provided additional insights into the abnormality of cancer metabolism.

In normal cells, glucose is catabolized to pyruvate, which can be later converted to acetyl-CoA to fuel the tricarboxylic acid cycle (TCA cycle, or Krebs cycle). TCA cycle generates NADH and FADH_2_ to provide mitochondrial respiratory chain with electrons for energy production. This is an effective energy production mode since each glucose molecule can produce up to 36 ATP, largely thanks to mitochondrial respiration. In normal cells, glycolysis is prioritized only when oxygen supply is limited. In contrast, cancer cells preferentially use glycolysis even in the abundance of oxygen^2^^,^^3^^,^^5^^,^^7^^,^^18^^-^^21^. This is why tumor glycolysis is often called “aerobic glycolysis”, or the Warburg effect, to distinguish from the normal anaerobic glycolysis of healthy cells.

However, cancer cells have to compensate for the 18-fold lower efficacy of energy generation (glycolysis only makes 2 ATP per glucose molecule consumed while mitochondrial respiration can produce up to 36 ATP for each glucose molecule catabolized). Part of the solution is to upregulate glucose transporters, especially Glut1, Glut2, Glut3, and Glut4, to uptake more glucose^5^^,^^22^^-^^24^. In fact, the increase in glucose uptake is a major feature distinguishing tumor cells from normal cells. This difference has been widely exploited in Positron Emission Tomography (PET) imaging modality using radiolabeled analogs of glucose such as ^18^F-fluorodeoxyglucose as a tracer to visualize tumors.

In addition, tumors remarkably elevate the expression of the majority of glycolytic enzymes. Major oncogenes such as Ras, Myc, and HIF-1α are reported to be master inducers of cancer glycolysis^3^^,^^5^^,^^24^. Many glycolytic enzymes are also upregulated in tumors because of elevated c-Myc and HIF-1α transcriptional activity and insufficient p53-mediated control. Indeed, c-Myc and HIF-1α are well recognized as two master inducers of glycolysis through direct or indirect transactivation of cancer glycolytic genes. These two transcription factors coordinate to promote the expression of key glycolytic enzymes such as *HK2, PFK1, TPI1, LDHA,* among others, in tumors^2^^,^^3^^,^^5^^,^^7^^,^^21^^,^^25^^,^^26^. In fact, most of glycolytic gene promoter areas contain consensus Myc and HIF-1α binding motifs. While HIF-1α is mainly functional in hypoxia, c-Myc is well known to promote its glycolytic target genes’ expression in normoxia. This coordination allows tumors to continuously drive glycolysis for supporting their rapid proliferation and accelerated biosynthesis^2^^,^^3^^,^^7^^,^^11^^,^^15^^,^^16^^,^^21^.

In contrast, p53 is known to suppress glucose uptake by directly inhibiting the transcription of glucose transporter Glut1 and Glut4^27^^,^^28^ and suppressing the expression of Glut3^28^. Glut3 is an NF-κB target gene and p53 is found to block NF-κB activation, thereby considerably reducing Glut3 transcription and expression^28^. p53 also induces the expression of *TIGAR* to slow down cancer glycolytic flux^29^^,^^30^. Fructose 2,6-bisphosphate is an important allosteric activator of PFK1, a major glycolytic enzyme. Fructose 2,6-bisphosphate is produced by PFK2 from fructose 1-phosphate. By converting fructose 2,6-bisphosphate back to fructose 1-phosphate, TIGAR significantly slows down tumor glycolysis^29^^,^^30^.

The interaction among p53, c-Myc and HIF-1α has a decisive impact on the status of cancer glycolysis^2^^,^^5^^,^^7^^,^^16^^,^^21^^,^^30^. Many studies have characterized the communication between these three master regulators of cancer glycolysis and how the balance among these factors control the status of cancer metabolism.

On the other hand, the way tumor cells process pyruvate, the end product of glycolysis, is also different from normal cells. In normal cells, most of pyruvate is converted to acetyl-CoA to fuel the TCA cycle. Some pyruvate is used to produce alanine or lactate. In contrast, pyruvate-to-lactate is a preferred reaction in tumor cells due to the upregulation of lactate dehydrogenase A (*LDHA*). This reaction is beneficial for cancer cells as it helps regenerate NADH to accelerate glycolysis^2^^,^^3^^,^^5^^,^^11^^,^^25^. Furthermore, lactate is secreted into tumor microenvironment via MCT4 transporter to fuel other cancer cells that do not have frequent access to nutrient supplies from blood stream. Lactate could be uptaken by MCT1 transporter and used by the TCA cycle for metabolism. The symbiosis of lactate-producing cancer cells and lactate-consuming tumor cells is an effective way for tumors’ adaptation to the diverse and constantly changing conditions in tumors, which is caused by the leaky and poorly formed tumor blood vessel network^7^^,^^31^^-^^33^. Furthermore, converting pyruvate to lactate also reduces reactive oxygen species’ levels, thereby diminishing the intracellular oxidative stress in cancer cells and promoting tumors’ survival^2^^,^^7^. Moreover, lactate also lowers the pH of extracellular microenvironment and facilitates the activity of metalloproteases for breaking down extracellular matrix. Thus, lactate is an inducer of cancer invasion and metastasis^34^^,^^35^.

Importantly, glycolysis provides cancer cells with not only energy but also necessary precursors for biosynthesis, which is similar to stem cells’ metabolic profiles. Several glycolytic metabolites such as glucose-6-phosphate, dihydroxyacetone phosphate, among others, could be diverted into other metabolic pathways. For instance, glucose-6-phosphate is often consumed by pentose phosphate pathway to synthesize nucleotides and NADPH (a major reducing agent important for redox homeostasis and drug detoxifying reactions). Dihydroxyacetone phosphate could be used for lipid synthesis, which is important for assembling new organelles and cells to promote tumor growth and proliferation. Metabolites from glycolysis are also important materials for amino acid production and macromolecules synthesis, which is required for active cell division and large-scale biosynthetic programs^2^^,^^3^^,^^5^^,^^7^^,^^16^^,^^36^^,^^37^. In addition to their metabolic function, glycolytic enzymes play active roles in promoting cancer survival, metastasis, invasion, chromatin remodeling, gene expression regulation, and other essential cellular processes^2^^,^^38^. Thus targeting glycolytic enzymes’ activities could be useful strategies for cancer therapy.

### Glutaminolysis

In addition to glycolysis, many tumors also rely on glutaminolysis to fuel their cellular bioenergetics and metabolism. Glutaminolysis is a series of biochemical reactions catabolizing glutamine into downstream metabolites such as glutamate, α-ketoglutarate. The products of glutaminolysis are very important to fuel the TCA cycle of tumors. The intermediates of TCA cycles could be used for the synthesis of lipid, cholesterol, amino acids and other essential metabolites. Moreover, NADH and FADH_2_ from the TCA cycle provide electrons for the electron transport chain of mitochondria to generate ATP. Thus, similar to glycolysis, glutaminolysis supplies cancer cells with not only ATP but also crucial precursors for continuous biosynthesis and accelerated proliferation^3^^,^^5^^,^^13^^,^^15^^,^^16^^,^^22^^,^^25^.

Glutaminolysis upregulation in tumors is mediated by c-Myc^4^^,^^9^^,^^13^^,^^39^. Multiple studies demonstrate that c-Myc promotes both glutamine uptake and the catabolic process of glutamine. In fact, c-Myc transactivates *ASCT2* and *SN2*, two important glutamine transporters on cellular membrane^9^^,^^40^. c-Myc also suppresses miR-23a/b to upregulate *GLS1* expression^41^^,^^42^. GLS1 is a major enzyme for glutaminolysis. Therefore, c-Myc is an important inducer of glutaminolysis in tumors. Interestingly, while promoting cancer metabolic reprogramming, c-Myc also renders cancer cells addicted to glutaminolysis, which opens a new therapeutic window to selectively suppress and eliminate cancer cells^9^^,^^13^^-^^15^^,^^39^^,^^43^. Therefore, targeting tumor glutaminolysis and c-Myc-induced-glutamine addiction is a promising anti-cancer metabolism therapy.

### Pentose phosphate pathway

Pentose phosphate pathway (PPP) is a classical metabolic pathway consisting of two branches. In the oxidative arm, PPP converts glucose-6-phosphate, a glycolytic intermediate, into ribulose-5-phosphate and generates NADPH. NADPH is then used for glutathione production, detoxification reactions, and biosynthesis of lipids as well as other macromolecules. The non-oxidative PPP branch involves reversible carbon-exchanging reactions with the final products as fructose-6-phosphate and glyceraldehyde-3-phosphate. These metabolites can participate in glycolysis and downstream metabolic pathways^44^. PPP is commonly viewed as a line of defense counteracting reactive oxidative stress and producing ribose-5-phosphate for nucleotide synthesis. However, new studies suggest that PPP has important impacts on various aspects of cancer, including proliferation, apoptosis, invasion, drug resistance, and metastasis^44^. These exciting findings unveil PPP as a potential target for anti-cancer metabolism therapies.

Rapidlyproliferating cancer cells constantly demand nucleotides and other materials for biosynthesis. Therefore, by providing NAPDH and pentose phosphate for nucleotide synthesis, PPP is important and frequently upregulated in many types of tumors^5^^,^^44^. In fact, the activity of glucose-6-phosphate dehydrogenase (G6PD), a major PPP enzyme, increases in proliferating cancer cells^45^. G6PD, transketolase (TK) and other PPP enzymes are elevated in multiple types of cancer and facilitated tumors’ accelerated proliferation^44^^,^^46^^,^^47^. In addition, G6PD also promotes cancer survival by producing NADPH, a key tool for tumor cells to defend against oxidative stress, chemotherapy-induced cytotoxic damage, as well as for promoting biosynthesis^44^. Hence, G6PD function is tightly controlled by the tumor suppressor p53. Indeed, p53 associates with G6PD and prevents this enzyme from forming active dimer complexes^48^. It is noteworthy that G6PD is directly transactivated by HIF-1α^49^. The function of G6PD is strictly regulated in normal cells but highly activated in cancer cells, making G6PD a strong oncogene candidate^44^. Interestingly, G6PD and TK functions are both suppressed by resveratrol^50^, suggesting the usage of this natural product in cancer treatment and prevention.

While normal cells frequently rely on the oxidative branch of PPP for ribose-5-phosphate production; cancer cells use both arms, e.g., oxidative and non-oxidative, of PPP to generate ribose-5-phosphate for nucleic acid synthesis^51^^-^^53^. Furthermore, cancer cells can use ribose-5-phosphate in both *de novo* and salvage pathways to synthesize nucleotides. These flexible metabolic programs help cancer cells effectively adapt to constantly changing nutritional conditions of tumor microenvironment.

In addition, PPP also protects tumor cells from apoptosis by counteracting oxidative stress and facilitating DNA damage repair. In fact, nonsteroidal anti-inflammatory medications induce apoptosis and shrinkage of colon carcinoma and polyps by regulating PPP^54^. Moreover, G6PD inhibitors, e.g., DHEA and 6-AN, promote apoptosis in mouse fibroblasts and PC-12 neural cells while overexpression of G6PD protects cells from H_2_O_2_-induced cell death^55^. Knocking down of G6PD also increases oxidative stress-mediated toxicity in melanoma cells^56^. The vital role of PPP in protecting cells from programmed cell death is additionally proven *in vivo* such as in stem cells and peripheral blood mononuclear cells of patients lacking G6PD^55^^,^^57^^,^^58^. Interestingly, the cytoprotective function of PPP is not limited to defending against reactive oxygen species but also expands to helping DNA damage repair. Indeed, upon DNA damage, ATM quickly activates G6PD functions to accelerate PPP for quenching reactive oxygen species, increasing nucleotide synthesis and enabling effective DNA repair. Therefore, knocking down G6PD significantly impairs DNA damage repair ability^59^^,^^60^. Some other studies describe the impact of PPP on regulation of autophagy^61^, but the molecular mechanism is still not completely understood.

Surprisingly, PPP also induces tumor angiogenesis. Leopold *et al*.^62^ and Pan *et al*.^63^ reported the crosstalk between G6PD and VEGF and tight association between G6PD and angiogenesis. These studies show that VEGF stimulate G6PD expression via Src signaling and G6PD is important for VEGF-induced-endothelial cell migration by increasing the phosphorylation of VEGR receptor Flk-1/KDR. G6PD also increases the proangiogenic activity of endothelial NO by providing NADPH and stimulates Akt-induced activation of endothelial nitric oxide synthase (eNOS)^62^.

PPP additionally promotes tumor resistance to chemotherapy and radiation by multiple mechanisms. First, PPP provides cancer cells with NAPDH, a potent anti-oxidative agent that protects cancer cells from reactive oxygen species-induced cell death caused by chemotherapy and radiation^44^; Second, PPP facilitates DNA damage repair by providing material for nucleotide synthesis; Third, by shifting cancer metabolism away from mitochondrial respiration, PPP lowers the intracellular concentrations of reactive oxygen species, thereby increasing tumor endurance and survival during chemotherapy and radiation treatment; Fourth, NAPDH derived from PPP, is an important element for glutathione (GSH) generation. GSH is frequently used in detoxification reactions, enabling cancer resistance to a variety of chemotherapeutic agents. GSH conjugation to these xenobiotics also facilitates the activity of MDR1 and MDR2 to discard cytotoxic substances. Therefore, increase in G6PD expression and PPP flux increase intracellular GSH levels and reduce drug accumulation in cancer cells^64^. However, there are still many exceptions where PPP neither significantly contributes to drug resistance nor promotes the effect of certain chemotherapeutic agents in several cancer cell lines. This complexity requires more study to fully elucidate the contribution of PPP in protecting cells from anti-cancer treatments^44^.

In short, PPP is an important metabolic pathway providing cancer cells with NADPH, ribose-5-phosphate and other essential intermediates. NAPDH is crucial for counteracting oxidative stress and biosynthesis reactions. Ribose-5-phosphate is a major element for nucleotide synthesis. Interestingly, the impact of PPP on cancer cells is well beyond oxidative defense. Indeed, PPP upregulation promotes cancer cell survival, angiogenesis, proliferation, invasion, metastasis, and resistance to radiation and chemotherapies. Therefore, elevated and active PPP enzymes, for instance, TKTL or G6PD, are frequently observed in malignant, aggressive, proliferative and drug-resistant cancer cells^44^. The new exciting discoveries about PPP open new therapeutic windows but also require more study to refine rational approaches for precise and effective targeting of this vital metabolic pathway in cancer cells.

### Mitochondrial biogenesis

Another major change in cancer metabolism is the enhancement of mitochondrial biogenesis. In contrast to conventional concepts, mitochondria play very important roles in cancer because these vital organelles are the nexus of many essential metabolic pathways^65^. Mitochondria are not only the energy generators but also the factories synthesizing many indispensable molecules for cellular biosynthesis, growth and proliferation. Moreover, mitochondria additionally control the redox balance and Ca^2+^ concentration, which is essential for cellular homeostasis^65^. Therefore, impairment of mitochondrial function or lack of mitochondrial biogenesis seriously suppresses tumorigenesis, tumor formation and growth^65^^-^^71^. Furthermore, in comparison with healthy and well differentiated cells, cancer cells frequently rewire their mitochondria to switch from a maximal energy production by mitochondrial electron transport chain to a well-adjusted balance among constant energy requirement, large-scale biogenesis programs and rapid cell proliferation^65^. Therefore, mitochondrial biogenesis and mitochondria are truly essential for tumor cells^65^. Hence, increase in mitochondria biogenesis is a significant advantage for cancer.

It is well established that c-Myc is a strong promoter of mitochondrial synthesis. In fact, c-Myc induces the expression of many nuclear-encoded mitochondrial genes. More importantly, c-Myc directly transactivates mitochondrial transcription factor A (TFAM). TFAM is a transcription factor that is indispensable for mitochondrial genes transcription and mitochondrial DNA replication^72^. In reality, TFAM promotes the right formation of mitochondrial transcription and replication complexes and facilitates the correct positioning of mitochondrial DNA for optimal gene transcription and proper mitochondrial DNA duplication^65^. As the synthesis of new mitochondrial components and replication of mitochondrial DNA are vital for *de novo* mitochondrial formation, c-Myc, indeed, plays a crucial role in elevating the number of mitochondria. As a consequence, lack of Myc expression and transactivational activity remarkably reduces mitochondrial mass as well as mitochondrial biogenesis, resulting in a severely suppressive impact on many metabolic pathways of cancer cells and tumorigenesis ultimately^72^.

### Lipid synthesis

Increase in lipid metabolism is another remarkable feature of cancer metabolism. Lipids are important building blocks of new organelles and cells. Lipid synthesis is a multiple step process involving several enzymes such as ATP citrate lyase (ACLY), acetyl-CoA carboxylase (ACC), fatty acid synthase (FASN), and stearoyl-CoA desaturase (SCD). This procedure starts with converting acetyl-CoA to malonyl-CoA by ACC. A series of condensation reactions by FASN results in saturated fatty acids. Fatty acids could be desaturated by SCD. Cancer cells frequently upregulate *de novo* fatty acid synthesis to satisfy their demands for lipids^73^^-^^75^. FASN elevation is observed in breast, prostate and other types of cancer^73^^,^^76^^-^^79^. FASN is a target gene of HIF-1α and frequently overexpressed in an Akt and SREBP1-dependent manner^80^. ACLY, often activated by Akt^81^, is indispensable for tumor transformation and formation both *in vitro* and *in vivo*^81^^,^^82^. ACC is also very important for tumorigenesis as inhibition of ACC stops cancer growth and induces apoptosis of prostate cancer cells^83^. Furthermore, cancer cells often have higher lipid accumulation in form of lipid droplets in relative to normal cells^84^.

Cholesterol synthesis, or the mevalonate pathway, is also an important aspect of lipid biosynthesis because cholesterol is a major component of membranes controlling the membrane fluidity and formation of lipid rafts. Cholesterol is vital for activation of Ras-Raf signaling pathway^85^ and deregulation of cholesterol synthesis is correlated with tumorigenic transformation^86^. Interestingly, statin-mediated inhibition of HMGCR, an important enzyme of the mevalonate pathway, considerably ameliorates the effectiveness of chemotherapies in acute myeloid leukemia^87^, hepatocellular carcinoma^88^, and other types of cancer through epigenetic pattern modification^89^.

The sterol regulatory element-binding proteins (SREBPs) are the main transcription factors controlling the expression of most of enzymes involved in fatty acid and cholesterol synthesis. SREBPs are helix-loop-helix 125 kDa proteins that require a protein cleavage at the endoplasmic reticulum for activation^73^. While SREBP1 controls fatty acid, triacylglycerol and phospholipid synthesis, SREBP2 regulates cholesterol generation^90^. SREBPs are controlled by tumor suppressors and oncogenes. AMPK, for instance, inhibits SREBP activation^91^ and suppresses ACC^91^, thereby keeping lipid synthesis in check. Loss of pRb upregulates SREBP1 and SREBP2, thereby activating Ras signaling^92^. p53 mutants, on the other hand, coordinates with SREBP to transactivate cholesterol-synthesizing enzymes^93^. Of note, SREBP1 and SREBP2 are often overexpressed in cancer^76^ and play an important role in cancer cell survival^94^.

At the organism level, excessive lipid synthesis contributes to tumorigenesis. It has been well documented that obesity increases the risk of cancer^73^. In fact, excessive lipid concentrations in liver and muscle cells induce insulin resistance by impairing insulin signaling and reducing glucose uptake. Insulin resistance forces pancreatic cells to secrete more insulin and insulin-like growth factors, which is very beneficial for cancer proliferation and survival^95^^-^^97^. Obesity also increases inflammation, which contributes to insulin resistance and tumorigenesis^98^. Dietary restriction may reverse these tumorigenic trends but in certain scenarios, especially when PI3K/Akt signaling is overactivated, the tumor-suppressing impact of dietary limitation decreased^99^. A possible explanation is that nutrient restriction may reduce the levels of circulating insulin and insulin-like growth factors. However, the constitutive activation of PI3K/Akt may compensate for that insulin signaling decrease^100^.

### Fatty acid oxidation

While glycolysis, glutaminolysis, fatty acid synthesis have been well characterized during the past few decades; fatty acid oxidation (FAO) still remains a little known metabolic pathway. However, recent studies have demonstrated the important contribution of FAO to tumorigenesis^101^.

Fatty acids are a rich energy source that can yield to up to two times more ATP than carbohydrates when needed. Fatty acids could be oxidized in mitochondria or by cytoplasmic lipophagy, a new fatty acid catabolic process^102^. FAO is a repeated multi-round process leading to the production of acetyl CoA, NADH, and FADH_2_ in each cycle. Acetyl-CoA can be imported into TCA cycle to generate more NADH and FADH_2_, which subsequently fuel mitochondrial respiration chain for ATP production. Acetyl-CoA can also fuel TCA cycle for synthesis of citrate. Citrate-derived isocitrate and malate can be respectively converted to α-ketoglutarate by IDH1 or pyruvate by malic enzyme (ME1)^102^. Both reactions generate NADPH, which plays a very important role in maintaining redox homeostasis, inducing cell survival, enabling xenobiotics detoxification and promoting biosynthesis for cell growth and division^103^. Of note, NAPDH is crucial for the function of many anabolic enzymes to sustain large-scale biosynthetic programs in many cancer cells.

NAPDH derived from FAO is very important for cancer cells to quench reactive oxidative stress. For instance, blocking glioma tumor’s FAO leads to rapid depletion of NADPH, surge of reactive oxidative species’ concentrations and increase in apoptosis^104^. NADPH produced by FAO is also relevant to the maintenance of hematopoietic stem cells because these cells are very sensitive and vulnerable to reactive oxidative stress. In fact, increased reactive oxygen species levels inhibit hematopoietic stem cells’ self-renewal and leads to cell differentiation^105^^-^^107^. Jeon *et al*.^108^ reported that LKB1-APMK regulates the balance between NADPH consumption by fatty acid synthesis and NAPDH production by FAO. In fact, AMPK blocks fatty acid synthesis in tumors by phosphorylating and inactivating acetyl-CoA carboxylase (ACC)^109^, antagonizing PPAR signal transduction^110^ and regulating CTP1C expression^111^. Therefore, AMPK is a potent inhibitor of fatty acid synthesis in cancer cells.

Needless for further emphasis, ATP is by large one of the most important molecules for cancer cells. Due to its rapid proliferation and accelerated activities, tumors are almost constantly in high demand for ATP. ATP is the most frequently used energy currency and a major material for phosphorylation reactions, an essential mode of cellular signal transduction and protein modification. ATP is also an indispensable element for DNA and RNA replication and repair. The function of MDR1 and other ABC pumps on cellular membrane, a major tumors’ line of defense against chemotherapy, absolutely requires ATP.

Recently, ATP production by FAO has been shown to prevent anoikis, a type of cell death due to loss of attachment to extracellular matrix although the molecular mechanism still remains unclear and warrants more study^103^^,^^112^. The Pandolfi group^113^ also reported that the promyelocytic leukemia (PML) protein induced FAO by activating peroxisome-proliferator-activated receptors (PPARs), leading to poor survival and clinical outcomes of breast cancer patients. Moreover, Tak Mak’s lab^111^ additionally found that carnitine palmitoyl-transferase 1 isoform C (CPT1C) is an oncogene that induces cancer growth, ATP production, FAO and confers resistance to mTORC1 inhibitors. CPT1 proteins mediate the import of fatty acids into mitochondria for FAO reactions. CPT1 links carnitine to fatty acids and transports the conjugated products (acyl-carnitines) into mitochondria. Therefore, the oncogenic property of CPT1C is a good example illustrating the potential of FAO in tumorigenesis.

FAO is also important in ensuring cancer cell survival in a manner that is independent of ATP production^101^. In fact, CPT1 proteins suppress the pro-apoptotic function of Bax and Bak by modulating the formation of mitochondrial permeability transition pores and reducing cytochrome c release^114^^,^^115^. The results from Samudio *et al*.^116^ and Vickers group^117^ additionally indicate that FAO can promote cancer cell survival by preventing a cytotoxic intracellular surge of fatty acid concentrations. On the other hand, several groups show that the increase in reactive oxygen species due to FAO-induced mitochondrial respiration could be harmful for leukemia cells. However, this toxicity could be resolved by upregulating uncoupling protein 2 and 3 (UCP2, UCP3) that effectively dissipate the gradient proton in mitochondria and decrease mitochondrial oxidative phosphorylation efficiency^118^.

Thus, fatty acid oxidation promotes cancer cell survival, and provides tumors with necessary energy and precursors. The new findings about FAO reveal fascinating understandings about cancer metabolic reprogramming and unveil very promising opportunities for anti-cancer therapeutic approaches. However, additional knowledge is needed to successfully develop effective therapies targeting this important catabolic process in cancer.

Interestingly, Hu *et al*.^119^ has recently completed a massive meta-analysis of over 2,500 microarrays including 22 types of cancer to compare the metabolic gene expression landscape of tumors relative to that of corresponding normal tissues. From this comprehensive transcriptomics analysis, three important observations have been reported: (1) despite the process of tumor evolution, there is still a significant degree of similarity in the gene expression metabolic profiles of tumors in comparison with those of the normal tissues where tumors originate; (2) the metabolic gene expression landscape across different types of tumors is heterogeneous. However, glycolysis, nucleotide synthesis, aminoacyl-tRNA synthesis, and pentose phosphate pathway are consistently upregulated and increasingly important in actively proliferating cancer cells; (3) hundreds of metabolic isoenzymes demonstrate remarkable and cancer-specific expression alterations, representing new significant therapeutic opportunities for anti-cancer metabolism therapies. These isoenzymes are important for cancer. Some enzymes such as isocitrate dehydrogenase and fumarate dehydratase, may even imitate or aggravate the impact of tumorigenic genetic mutations^119^.

In short, metabolic reprogramming is an important cancer hallmark characterized by the upregulation of glycolysis, glutaminolysis, lipid metabolism, mitochondrial biogenesis, pentose phosphate pathway as well as other biosynthetic and bioenergetic pathways. These cancer metabolic programs provide tumor cells with not only necessary energy but also crucial materials to support large-scale biosynthesis, rapid proliferation, survival, invasion, metastasis and resistance to anti-cancer therapies. Therefore, exploiting the unique features of cancer metabolism for cancer detection, treatment and monitoring is a very promising trend in cancer therapeutics, diagnosis and prevention.

## Cancer metabolism and diagnostic imaging

The distinguished features of cancer metabolism have been extensively exploited for initial diagnosis, staging disease, monitoring tumor responses to therapies, and detecting cancer recurrence^120^. Therefore, nowadays, metabolic molecular imaging plays an indispensable role in clinical oncology. These diagnostic methods are non-invasive and can accurately detect the changes in selective biologic processes of tumors compared to normal surrounding tissues both at the initial tumor sites and metastatic locations over an extended period of time. The information provided by advanced imaging modalities such as PET, magnetic resonance spectroscopy imaging (MRSI), magnetic resonance imaging (MRI), is very valuable for cancer detection, prevention, and treatment^120^.

### Positron emission tomography

PET is frequently combined with X-ray computed tomography (CT) to provide detailed information about cancer and anatomic locations of tumors. PET measures the signals of radiolabeled tracers taken up by cancer cells. PET is safe and widely used in clinics because the small amount of imaging probes doesn’t interfere with normal physiological processes. ^18^F-fluoro-2-deoxyglucose (FDG) is the most commonly used PET imaging material. Since most of tumors have a high glycolytic flux, elevated glucose uptake and increased hexokinase function, they will often have higher FDG signals relative to normal tissues. After being imported into tumor cells, FDG is phosphorylated by hexokinase but phosphorylated FDG cannot be further catabolized by glycolytic pathway. Therefore, phosphorylated FDG molecules are accumulated in tumors and can be detected by PET scanners. In clinics, FDG-PET scan is commonly used for determining cancer stages, identifying cancer recurrence and assessing tumor response to anti-cancer therapies^121^^,^^122^.

In addition to upregulated glycolysis, other patterns of cancer metabolism are also used for molecular oncology imaging using PET scan. Choline, for example, is frequently absorbed by tumor cells and used for new cellular membrane biosynthesis, an important process for cell division. Therefore ^11^C and ^18^F radiolabeled choline tracers have been successfully applied in hepatocellular carcinoma, lung, brain, and prostate cancer diagnosis^123^^-^^126^. Similarly, 3'-deoxy-3'-^18^F-fluorothymidine is often used to monitor cancer cell proliferation *in vivo*. 3'-deoxy-3'-^18^F-fluorothymidine is a thymidine analog and frequently phosphorylated by thymidine kinase 1. This enzyme is highly active in rapidly dividing cells, e.g., tumor cells, especially in S phase. Thus, 3'-deoxy-3'-^18^F-fluorothymidine PET can identify and measure tumor malignancy, tracking the efficacy of anti-cancer therapies^127^. Many other tracers are also used in PET imaging modality to monitor specific biological processes of tumors. For instance, ^68^Ga-DOTATOC, a high-affinity ligand for somatostatin receptor 2, is used to detect neuroendocrine cancer masses^128^. 16-α-^18^F-fluoro-17β-estradiol is used to quantify ERα and ERβ expression^129^. Tumor angiogenesis and the effectiveness of anti-angiogenic therapeutic agents are measured by tracers containing arginine-glycine-aspartic acid-peptide ligands. These ligands associate with α_v_β_3_ integrin whose expression is elevated on newly formed blood vessels^130^. Nitroimidazole is also exploited to image hypoxic areas where tumors are frequently located^131^.

In summary, PET with radiolabeled metabolic tracers is certainly a valuable and powerful imaging method with vast applications in clinical oncology. This diagnostic modality is continuously improved and more advanced tracers are in development. However, radiation is still a major concern for PET and its tracers. The radiation containment and safety are also other significant issues for PET application in clinics^120^. In addition, a complete understanding about cancer metabolic patterns and bioenergetics programs is crucial to continuously innovate metabolic tracers-based PET scan imaging.

### The combination of MRI and MRSI

MRI and MRSI are often combined in clinical oncology diagnostics because ^1^H MRSI is easily compatible with currently available MRI scanners in clinics^132^^-^^134^. ^1^H MRSI has a high sensitivity and could be applied on a number of tracers^120^. During the past few years, MRSI has made significant advances and rapidly become a reliable imaging modality. A number of ^1^H tracers have been successfully developed. For instance, ^1^H choline-containing metabolites are employed to measure tumor malignancy. Choline is an important component of cellular membrane. Higher choline concentrations are detected in aggressive and malignant tumors in comparison with benign and normal tissues^135^^,^^136^. In fact, many breast tumors contain a large amount of choline while benign tumor masses often have low levels of choline^135^^,^^136^. Since the accumulation of choline is associated with increased cell proliferation in brain, breast, cervical and prostate cancers^133^^,^^137^^-^^139^, choline availability could be used as a marker for predicting tumor histologic grade, aggressiveness, and even response to anti-cancer therapies with low unspecific detection rates^120^^,^^139^. Moreover, as brain tumors often have increased choline concentrations and diminished levels of N-acetyl aspartate, the ratio of choline/N-acetyl aspartate has been used to evaluate the aggressiveness of several types of brain tumors^140^^-^^142^. Choline/creatinine ratio measurement is also a valuable indicator of oligodendroglial cancer grade^143^.

^13^C tracers are emerging important diagnostic probes although their application is still at early stages. Recently, Nelson *et al*.^144^ reported a successful preclinical study and phase I clinical trial results with 31 prostate cancer patients. This is a pioneer project examining the applicability and safety of hyperpolarized ^13^C pyruvate tracers to monitor and evaluate the metabolic changes, especially ^13^C pyruvate-to-^13^C lactate flux, of prostate tumors in patients. This technique enabled a 10,000-fold increase in signals compared to regular MRI. Results were very promising with excellent safety profiles and accurate detection of ^13^C pyruvate-to-^13^C lactate flux in tumor areas that were subsequently proven by biopsy-based pathological and histological analyses. The success of this pioneer study paves a new way for non-invasive, safe, precise, and sensitive cancer diagnosis as well as tumor monitoring. A number of new types of ^13^C metabolic tracers are under development and will certainly play a major role in cancer detection and imaging in future.

Poor spatial resolution used to be a challenge for MRSI^133^^,^^138^^,^^145^^,^^146^, but new advances and ongoing technological improvements are addressing this limiting factor, making MRSI a promising adjunct to MRI. Combining conventional MRI with MRSI will enable accurate, safe and non-invasive characterization of tumors. This new diagnostic strategy is especially important when collecting lesion biopsies is risky, painful and difficult. Thus, in future, this new combinatory imaging modality will reduce patients’ discomfort, concern, risk, pain, and avoid unnecessary invasive diagnostic procedures while increasing the accuracy, reliability and sensitivity of diagnosis^120^.

In summary, diagnostic imaging plays a crucial role in cancer detection and treatment. Exploiting the unique features of cancer metabolism is a very promising direction for developing novel diagnosis methods to accurately detect cancer lesions even at early stages and precisely monitor tumors’ responses to therapies.

## Therapeutic implications

Given the vital role of metabolic reprogramming for tumorigenesis, targeting cancer bioenergetics is a very promising and rapidly rising direction for anti-cancer therapy development nowadays. Many compounds have been developed to selectively and effectively inhibit metabolic enzymes that are important for tumors. These inhibitors are currently at various stages of clinical trial process and we expect to see them in clinics within five to ten years from now.

One of the most common trends in anti-cancer metabolism therapies is to inhibit enzymes that are exclusively or mostly expressed or used in tumor cells. This therapeutic strategy would effectively eliminate tumors while minimizing damage to normal cells. Several groups have successfully developed inhibitors for Glutaminase 1 (GLS1), a glutaminase isoform that is highly upregulated in cancer cells, and proved the efficacy of blocking GLS1 in cancer treatment^147^^,^^148^. This tactic bases on previous studies showing a significant dependence of c-Myc-overexpressing cancer cells on glutaminolysis^9^^,^^11^^-^^15^^,^^25^^,^^149^. Similarly, modulating the activity of PKM2, a glycolytic enzyme that is frequently elevated in tumors, is also a promising therapy^150^^,^^151^. Fatty synthase (FASN) is important for palmitate synthesis and this enzyme’s expression is elevated in many tumors. Therefore, several groups have developed FASN inhibitors to target tumorigenesis^75^^,^^152^. Many inhibitors for HIF and HIF targets, for instance monocarboxylate transporter MCT4 and carbonic anhydrase IX (CAIX) are also potential anti-cancer drugs in future^153^^-^^156^. Similarly, MCT1 and carbonic anhydrase XII are targets of great potential^153^^,^^154^. MCT1 and MCT4 suppressors inhibit cancer growth *in vitro* and *in vivo* and invasion *in vitro*^157^^-^^160^. In fact, interfering with lactate transport by MCT1 and MCT4 inhibitors has been shown to induce tumor cell starvation and subsequent apoptosis^158^.

Blocking lactate production using dichloroacetate (DCA) shows promising results with minor side effects in early phase clinical trials, especially in glioblastoma patients^161^^,^^162^. DCA is found to promote pyruvate-to-acetyl-CoA flux and reduce pyruvate-to-lactate conversion, thereby inducing tumor shrinkage and apoptosis *in vivo*^161^^-^^163^. Clinical trial data show that DCA also suppresses tumor angiogenesis, blocks HIF1-α signaling and activates p53 in glioblastoma multiforme patients^161^. Initial studies additionally find that DCA inhibits pyruvate dehydrogenase kinase 1 (PDK1) activity and thereby activating the function of pyruvate dehydrogenase 1 (PDH1), an important enzyme catalyzing the pyruvate-to-acetyl-CoA biochemical reaction^162^^,^^163^. However, more and larger clinical studies are needed to fully elucidate the mechanism of action of this interesting compound and further evaluate its efficacy in cancer patients.

Glycolysis inhibitors are also of interest for many groups and pharmaceutical companies. For instance, FX11, a selective suppressor of lactate dehydrogenase A (*LDHA*) activity, was tested by Le *et al*.^164^ and is currently studied by National Cancer Institute’s Experimental Therapeutics Program (NExT). 2-deoxyglucose (2-DG) is among the most advanced cancer metabolism inhibitors in clinical trials (Phase II). 2-DG reversibly inhibits hexokinase to block glycolysis. 2-DG usage in combination with radiation demonstrates a good safety profile and slightly improves survival of glioblastoma multiforme patients^165^^,^^166^. However, the effects of 2-DG may be limited by high concentration of glucose because 2-DG-mediated inhibition of hexokinase is reversible.

Inhibiting mutant isocitrate dehydrogenase 1 (IDH1) and isocitrate dehydrogenase 2 (IDH2) is a remarkable therapeutic approach because these mutant enzymes have distinct activities compared to normal IDH1 and IDH2 in the healthy cells. On the other hand, metformin, a common anti-diabetics medication, has demonstrated very promising impact in cancer treatment. It is well known that metformin inhibits mitochondrial complex I of liver cells, thereby decreasing ATP production. Lack of ATP subsequently stimulates LKB1-AMPK pathway and blocks gluconeogenesis, leading to lower blood glucose concentrations, improved sensitivity to insulin and diminished insulin production^167^. It is currently unclear whether metformin improves cancer patient clinical outcomes by lowering blood glucose levels and insulin/insulin-like growth factors generation or by directly targeting cancer cells. Nevertheless, the usage of metformin has been well documented to ameliorate cancer patient survival^168^^,^^169^ and metformin are harmful for cancer stem cells^170^. Clinical trials testing the impact of metformin on cancer in patients are ongoing (**Table 1**).

**Table 1:** List of several potential anti-cancer metabolism compounds

Compound	Pathway target	Mechanism of action	Status	Source (if available)
2-Deoxyglucose	Glycolysis	Reversibly inhibiting hexokinase	Ongoing clinical trials with promising initial data	
3-Bromopyruvate	Glycolysis	Inhibiting hexokinase and other glycolytic enzymes	Preclinical	
Phloretin	Glucose transport	Glucose transporter Glut 1 and Glut 4	Preclinical	
Lonidamine	Glycolysis	Hexokinase	Clinical trials	
3PO	Glycolysis	Inhibiting activation of PFK1 by targeting PFKFB3 (phosphofructose kinase 2)	Preclinical	Advanced Cancer Therapeutics
BPTES	Glutaminolysis	Inhibiting glutaminase 1, a glutaminolytic enzyme frequently upregulated in many tumors	Preclinical	
968	Glutaminolysis	Inhibiting glutaminase 1, a glutaminolytic enzyme frequently upregulated in many tumors	Preclinical	Cornell University
IDH1/2 inhibitors	Blocking IDH1/2 altered function	Suppressing the function of mutant IDH1 and IDH2		Agios Pharmaceuticals
PKM2 inhibitors	Glycolysis	Inhibiting PKM2 function and reducing pyruvate synthesis		Agios Pharmaceuticals
PKM2 activators	Biosynthesis	Activating PKM2 to reduce glycolytic intermediates shunt to biosynthetic pathways		Agios Pharmaceuticals
Dichloroacetate	Lactate production	Blocking PDK1 activity thereby increasing PDH1 function and facilitating pyruvate-to-acetyl coA reaction to fuel TCA cycle and mitochondrial respiration	Phase I completed with promising results in glioblastoma multiforme patients	
Metformin	Energy production pathways	Inhibiting mitochondrial complex I and lipid and protein synthesis, modulating glycolysis, decreasing glucose supply, insulin and insulin-like growth factor signaling availability for tumor cells	Ongoing clinical trials for cancer	
FX11	Lactate production	Inhibiting function of Lactate Dehydrogenase A thereby blocking lactate production in cancer	Preclinical	John Hopkins University and University of New Mexico
AZD-3965	Lactate transport	Blocking MCT1 activity, thereby inhibiting lactate transport	Clinical trials Phase I ongoing in UK	AstraZeneca
L-asparaginase	Asparagine and glutamine availability	Promote asparagine and glutamine degradation, thereby cutting the supply of these amino acids for cancer cells	Approved for usage in leukemia. Effective therapy	

Importantly, there is also an urgent need to develop effective inhibitors to target the key inducers of cancer metabolic reprogramming such as c-Myc and Ras. Ras mutations and c-Myc upregulation are frequent in many common types of cancer and these dysregulations are major drivers of tumorigenesis and resistance to therapies^171^^,^^172^. However, despite our relentless efforts, effectively and directly inhibiting Ras and c-Myc still requires a lot more study because these two proteins are currently undruggable targets. Interestingly, several preclinical research projects show that targeting metabolic enzymes significantly inhibits tumors carrying Ras mutation and c-Myc overexpression^9^^,^^173^. In fact, suppressing glycolysis and glutaminolysis remarkably antagonizes the growth of tumors bearing those genetic alterations^9^^,^^164^^,^^174^^,^^175^. These observations imply a new way to treat tumors carrying genetic mutations that can’t be directly targeted.

Another striking example of successful anti-cancer metabolism therapies is L-asparaginase. L-asparaginase mediates deamination reactions to degrade asparagine into aspartic acid^176^, thereby reducing asparagine availability to cancer cells and suppressing their growth^177^^,^^178^. This therapy is very effective for acute lymphoblastic leukemia (ALL) and related leukemia subtypes because ALL cells are unable to synthesize asparagine^179^. Therefore, these cancer cells have to rely on extracellular asparagine sources and become very vulnerable when asparagine supplies are limited.

However, lymphocytes, especially T cells, have similar metabolic programs as those in tumor cells. For instance, lymphocytes also depends on glutamine metabolism^180^, suggesting that systematically targeting glutaminolysis for cancer treatment may severely affect adaptive immune responses and also innate immunity to a certain degree. These metabolic similarities between cancer cells and lymphocytes explain why many agents targeting cancer metabolism are also strong immunosuppressants. For instance, cyclosporine, a potent anti-cancer drug that inhibits mTOR, significantly suppresses immune system. Suppressor of nicotinamide phosphoribosyltransferase (NAMPT), an enzyme responsible for nicotinamide adenine dinucleotide (NAD^+^) regeneration, is poisonous to lymphocytes^181^. In fact, early clinical trials data show that FK866, a NAD^+^synthesis inhibitor, leads to mild lymphopenia and severe thrombocytopenia^182^.

These findings suggest that immunosuppression could be a challenge for therapies designed to target cancer cells’ bioenergetics as the Achilles’ heel of tumors. Nevertheless, there is still a significant therapeutic window for anti-cancer metabolic therapies. We just need to identify the key differences in the bioenergetics patterns of tumors and those of healthy cells in order to optimize our therapies for precisely inhibiting the unique metabolic targets in cancer cells. A significant example is to use BPTES to selectively block GLS1, a glutaminase enzyme isoform that is crucial for cancer cells and specifically upregulated in tumors^147^^,^^148^.

## Conclusion

**Figure 1:**
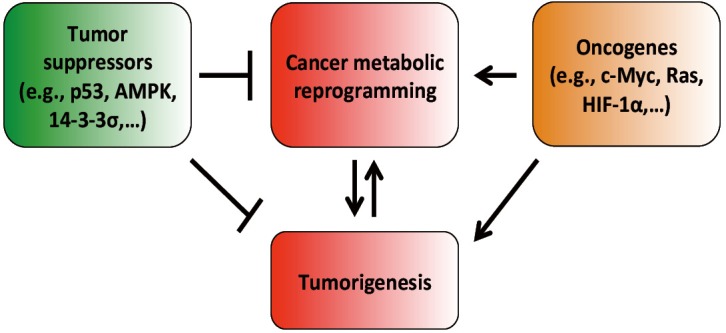
The impacts of tumor suppressors and oncogenes on cancer metabolic reprogramming, an important cancer hallmark. Cancer metabolic alterations are the results of oncogene activation and mutant metabolic enzymes. Cancer metabolic reprogramming promotes tumorigenesis by facilitating and enabling rapid proliferation, survival, invasion, metastasis, resistance to therapies and other central cellular processes of tumorigenesis. On the other hand, as tumorigenesis advances, cancer cells acquire more mutations and changes that further enhance metabolic reprogramming and, in turn, accelerate tumor growth, proliferation and progression. Tumor suppressors, for instance, p53, and AMPK, exert their suppressive regulation on cancer metabolic alterations by blocking the function, activation and expression of essential cancer metabolic genes. Our recent results also show that 14-3-3σ, a downstream target gene of p53, effectively opposes and reverses cancer metabolic reprogramming. Our data indicate that 14-3-3σ accelerates the degradation of c-Myc, an important transcription factor promoting cancer metabolic reprogramming^183^. In contrast, oncogenes such as c-Myc, HIF-1α, Ras, and Akt are major inducers of tumor bioenergetics alterations by upregulating the expression or activation of key metabolic enzymes such as HK2, GLS1, LDHA, among others. The balance between tumor suppressors and oncogenes has a decisive impact on the status of cancer metabolism.

**Figure 2:**
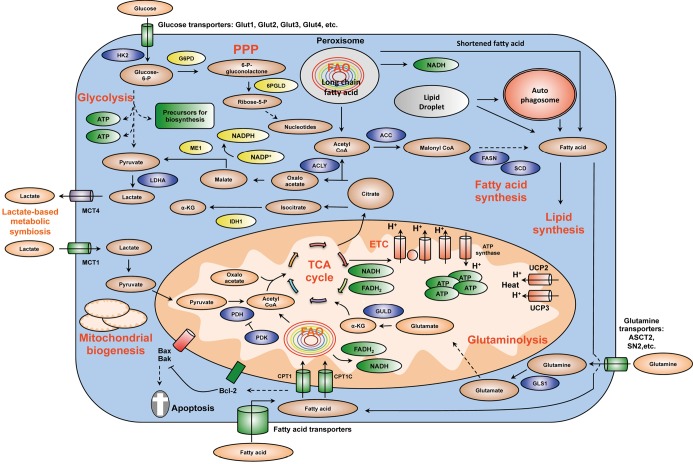
Summary of key changes in cancer metabolic reprogramming. Cancer metabolic reprogramming is characterized by enhanced glycolysis, PPP, lipid metabolism, glutaminolysis, mitochondrial biogenesis, among others. These pathways provide cancer cells with not only essential energy but also important precursors to support large-scale biosynthesis, rapid proliferation, continuous growth, tissue invasion, metastasis, survival and resistance to anti-cancer therapies. For instance, glycolysis generates 2 ATP per glucose consumed and provides materials for PPP and other biosynthetic programs. Similarly, PPP supplies tumors with ribose-5-phosphate and NADPH. Ribose-5-phosphate is a major element for nucleotide synthesis, which is used in DNA replication, RNA synthesis, and DNA damage repair, among others. NADPH is a key line of defense counteracting oxidative stress and a crucial metabolite for a number of biosynthesis reactions. NADPH is produced by 4 biochemical reactions mediated by G6PD, 6PLGD, ME1 and IDH1. In addition, fatty acid synthesis is indispensable for formation of new cellular membranes and proliferation. A number of fatty acid synthesis enzymes such as ACC, ACLY and FASN are upregulated or activated by oncogenes such as c-Myc, HIF-1α, Akt, among others. On the other hand, FAO is also important for cancer cells because it generates energy, NADPH and other necessary metabolites. Fatty acids are imported into mitochondria by CPT1 and oxidized to generate acetyl-CoA. Acetyl-CoA fuels the TCA cycle to generate NADH and FADH_2_. The latter metabolites donate electrons to mitochondrial ETC for ATP generation. CPT1 also antagonizes Bax and Bad-mediated apoptosis by preventing the formation of mitochondrial membrane transition pores and reducing cytochrome c release. Citrate produced by the TCA cycle can be transported from mitochondria to cytosol. Cytosolic citrate is used in a number of reactions to produce acetyl-CoA, oxaloacetate and isocitrate. These metabolites are important for lipid synthesis, NAPDH production, and many other central cellular processes. Mitochondrial biogenesis is also a striking feature of cancer metabolic reprogramming. Mitochondria are not only the energy generators but also the factories for synthesizing many essential metabolites for cancer growth, proliferation and metastasis. In addition, the metabolic lactate-based symbiosis is another remarkable characteristic of cancer metabolism. Cancer cells frequently upregulate LDHA to facilitate the conversion of pyruvate to lactate. Lactate is then secreted to tumor microenvironment via MCT4 transporters and can be taken by neighboring cancer cell thanks to MCT1 importers. Lactate is thereafter used for other metabolic pathways in tumors. This metabolic symbiosis facilitates the survival of cancer cells in harsh conditions. Thus, metabolic reprogramming is a major cancer hallmark. It is characterized by the upregulation of a number of inter-connected metabolic pathways providing cancer cells with vital energy and metabolites. This metabolic plasticity is essentially important because it allows cancer cells to effectively and rapidly adapt to the rapidly changing conditions of tumor microenvironment. In addition, the flexibility of cancer bioenergetics also enables rapid proliferation, continuous growth, invasion, metastasis and resistance to anti-cancer therapies. Therefore, further knowledge about cancer metabolic reprogramming is very important for successful development of precise and efficacious anti-cancer metabolism therapies. Dashed arrows indicate indirect effects or multi-step processes. Abbreviations: HK2, hexokinase 2; LDHA, lactate dehydrogenase A; G6PD, glucose-6-phosphate dehydrogenase; 6PGLD, 6-phosphogluconate dehydrogenase; ACC, acetyl-CoA carboxylase; ACLY, ATP citrate lyase; FASN: fatty acid synthase, SCD, stearoyl-CoA desaturase; CPT, carnitine palmitoyltransferase; CPT1C, carnitine palmitoyltransferase 1C; PDH, pyruvate dehydrogenase; PDK, pyruvate dehydrogenase kinase; UCP, uncoupling proteins; MCT, monocarboxylic acid transporter; ME1, malic enzyme; IDH1, isocitrate dehydrogenase1; GLS1, glutaminase; GLUD, glutamate dehydrogenase; FAO, fatty acid oxidation; ETC, electron transport chain; PPP, pentose phosphate pathway; TCA, tricarboxylic acid cycle; α-KG, alpha-ketoglutarate.

**Figure 3:**
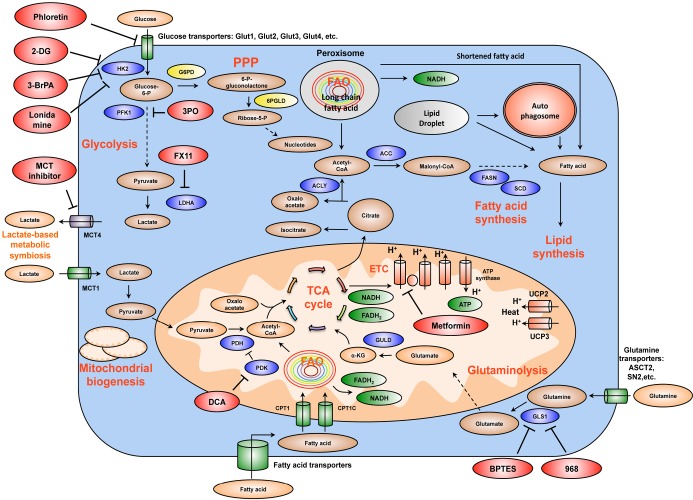
Summary of the mechanism of several important drug candidates for anti-cancer metabolism therapies. Phloretin inhibits the import of glucose, a major source of nutrient for cancer cells. 2DG, 3BrPA, and Lonidamine inhibit HK2, a rate-limiting step of glycolytic pathway. 3PO blocks PFK1 activation by inhibiting PFKFB3 (PFK2). FX11 selectively inhibits LDHA, a major metabolic enzyme of cancer. BPTES and 968 suppress the function of GLS1. GLS1 is a glutaminolytic enzyme that is highly and selectively upregulated in cancer. DCA inactivates PDH kinase (PDK), thereby increasing PDH activity and enhances the conversion of pyruvate to acetyl-CoA and decreases cancer glycolysis. Metformin blocks energy production of cancer cells by inhibiting mitochondrial complex I, suppresses lipid and protein synthesis, modulates glycolysis. At the organism level, by lowering blood glucose concentration, metformin decreases glucose supply, as well as insulin and insulin-like growth factor signaling availability for tumor cells. MCT inhibitors impair the metabolic lactate-based symbiosis of cancer cells. Many other anti-cancer metabolism compounds are under development. Targeting cancer metabolism is a very promising direction for anti-cancer therapies. It is expected that inhibitors of tumor metabolism will play an important role in clinical oncology within five or ten years. These medications could be used alone or in combination with other current anti-cancer therapies to increase efficacy. Abbreviations: 2DG, 2-deoxyglucose; 3BrPA, 3-bromopyruvate; HK2, hexokinase 2; PFK1, phosphofructose kinase 1; LDHA, lactate dehydrogenase A; GLS1, glutaminase 1; DCA, dicholoroacetate; PDH, pyruvate dehydrogenase; PDK, pyruvate dehydrogenase kinase; MCT, monocarboxylic acid transporter.

Metabolic reprogramming is a major hallmark of cancer, which is characterized by upregulated glycolysis, glutaminolysis, lipid metabolism, pentose phosphate pathway, mitochondrial biogenesis, among others. These metabolic programs provide cancer cells with not only energy but also vital metabolites to support large-scale biosynthesis, continuous proliferation and other major processes of tumorigenesis. Potent oncogenes as c-Myc, HIF1α, Ras and PI3K/Akt are important promoters of cancer metabolic alterations. In contrast, major tumor suppressors such as p53 and LKB1/AMPK antagonize those changes and keep cellular metabolism in check (**Figure 1** and **Figure 2**). Rfewiring metabolism is very beneficial for tumor survival, invasion, metastasis, growth, angiogenesis, proliferation and resistance to anti-cancer therapies. Although there is still much to study and discover, recent remarkable advances in this field have unveiled exciting therapeutic windows to precisely and effectively target cancer metabolism and bioenergetics (**Figure 3**). It is expected that anti-cancer metabolism therapies will play an important role in clinical oncology within five or ten years.

However, the efficacy of anti-cancer metabolism therapies will need to be carefully evaluated because cancer cells are well known for their metabolic plasticity and heterogeneity^1^^,^^2^^,^^11^^,^^21^^,^^119^^,^^184^. That may enable tumors to bypass certain inhibition mediated by therapeutic agents. Furthermore, as we have seen during the past decades, inhibiting individual enzymes or blocking single pathways seldom leads to effective cancer treatment. Therefore, it is highly likely that anti-cancer metabolism approaches need to be combined with other therapies to improve therapeutic effects and clinical outcomes. Further understanding about cancer metabolic reprogramming is certainly needed for effective therapy development. Nevertheless, exploiting the unique features and weakness of tumor metabolism for cancer treatment, detection and monitoring is clearly a very promising direction.
